# A Grey Wolf Optimizer for Modular Granular Neural Networks for Human Recognition

**DOI:** 10.1155/2017/4180510

**Published:** 2017-08-14

**Authors:** Daniela Sánchez, Patricia Melin, Oscar Castillo

**Affiliations:** Tijuana Institute of Technology, Tijuana, BC, Mexico

## Abstract

A grey wolf optimizer for modular neural network (MNN) with a granular approach is proposed. The proposed method performs optimal granulation of data and design of modular neural networks architectures to perform human recognition, and to prove its effectiveness benchmark databases of ear, iris, and face biometric measures are used to perform tests and comparisons against other works. The design of a modular granular neural network (MGNN) consists in finding optimal parameters of its architecture; these parameters are the number of subgranules, percentage of data for the training phase, learning algorithm, goal error, number of hidden layers, and their number of neurons. Nowadays, there is a great variety of approaches and new techniques within the evolutionary computing area, and these approaches and techniques have emerged to help find optimal solutions to problems or models and bioinspired algorithms are part of this area. In this work a grey wolf optimizer is proposed for the design of modular granular neural networks, and the results are compared against a genetic algorithm and a firefly algorithm in order to know which of these techniques provides better results when applied to human recognition.

## 1. Introduction

In this paper, a grey wolf optimizer for modular granular neural networks (MGNN) is proposed. The main goal of this optimizer is the design of modular neural networks architectures using a granular approach and to evaluate its effectiveness, these modular granular neural networks are applied to one of the most important pattern recognition problems, human recognition. For a long time human recognition has been a widely studied area, where its study mainly lies in finding those techniques and biometric measures that allow having a trustworthy identification of persons to protect information or areas [1, 2]. Some of the most used biometric measures are face [3, 4], iris [5], ear [6, 7], voice [8], vein pattern [9], hand geometry [10], signature [11], and gait [12], among others.

On the other hand, within the most used techniques are those that belong to the soft computing category such as artificial neural networks [13, 14], fuzzy logic [15], computational vision [16], granular computing [17, 18], data mining [19], and evolutionary computation [20, 21]. Within the evolutionary computation area, bioinspired algorithms are found to be one of type of method. The already well-known genetic algorithm (GA) [22, 23], ant colony system (ACO) [24], particle swarm optimization (PSO) [25], bat algorithm (BA) [26], grey wolf optimizer (GWO) [27], harmony search (HS) [28], gravitational search algorithm (GSA) [29], and firefly algorithm (FA) [30, 31], just to mention a few, belong to this category.

It is important to mention that some soft computing techniques such as neural networks and fuzzy logic combined with a bioinspired algorithm can allow achieving better performance when they are individually used. When two or more techniques are combined the resulting system is called hybrid intelligent system [7, 32]. In this paper a hybrid intelligent system is proposed using modular neural networks (MNN), granular computing (GrC), and a grey wolf optimizer (GWO). The optimization of artificial neural network (ANN) using a grey wolf optimizer was already proposed in [33–36]. These works applied their methods to classification and function-approximation, where optimal initials weights of a neural network are sought using the grey wolf optimizer.

A modular neural network is an improvement of the conventional artificial neural network, where a task is divided into subtasks and an expert module learns some of these subtasks without communication with other modules; this technique allows having systems resistant to failures and works with a large amount of information. Usually this kind of networks has been used for human recognition based on biometric measures, classification problems, and time series prediction [40]. On the other hand, granular computing defines granules as classes or subsets used for complex applications to build computational models where a large amounts of data and information are used [19, 41, 42]. In this work granular computing is applied to perform granulation of information into subsets that also define number of modules of a modular neural network; the combination of modular neural networks and granular computing was already proposed in [7, 37, 38], where the advantages of modular granular neural networks over conventional neural networks and modular neural networks were widely demonstrated. In [7], the modular granular neural network architectures were designed using an improvement of a genetic algorithm, a hierarchical genetic algorithm (HGA), where the main differences between them are the control genes in the HGA that allow activating and deactivating genes allowing solving complex problems. That design consisted in optimization of number of modules (subgranules), percentage of data for the training phase, learning algorithm, goal error, and number of hidden layers with their respective number of neurons. In [38], a firefly algorithm was proposed for MGNN optimization using an experts submodules for each division of image. In [37], also modular granular neural network architectures were designed but using a firefly algorithm and without an expert submodule for each division of image. In this work, the design of MGNN architecture is performed and applied to human recognition based on ear, face, and iris, but using a grey wolf optimizer, statistical comparisons are performed to define which of these optimization techniques is better to perform optimization of MGNNs.

This paper is organized as follows. In Section 2, the proposed method is described. The results achieved by the proposed method are explained in Section 3. In Section 4, statistical comparisons of results are presented. Finally, conclusions are given in Section 5.

## 2. Proposed Method

The proposed hybrid intelligence method is described in this section; this method uses modular neural networks with a granular approach and their architectures are designed by a grey wolf optimizer.

### 2.1. General Architecture of the Proposed Method

**Figure 1:**
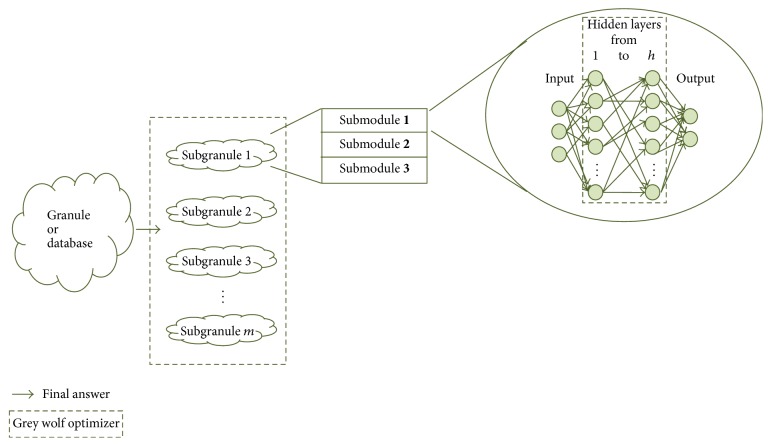
The general architecture of proposed method.

The proposed method uses modular granular neural networks, this kind of artificial neural network was proposed in [7] and [37], and their optimization were performed using, respectively, a hierarchical genetic algorithm and a firefly algorithm. In this work, the optimization is performed using a grey wolf optimizer and a comparison among HGA, FA, and GWO is performed to know which of these techniques is better for MGNN optimization. As a main task, the optimization techniques have to find the number of subgranules (modules), and as a preprocessing process each image is divided into 3 regions of interest; these regions will be described later. In Figure 1, the granulation process used in this work and proposed in [7] is illustrated, where a database represents a whole granule. This granule can be divided into “*m*” subgranules (modules), this parameter (*m*) can have up to a certain limit set depending on the application, each of these subgranules can have different size for example, when this granulation is applied to human recognition, and each granule can have different number of persons that the corresponding submodules will learn. The grey wolf optimizer in this work performs optimization of the granulation and hidden layers and other parameters described later.

#### 2.1.1. Description of the Grey Wolf Optimizer

**Figure 2:**
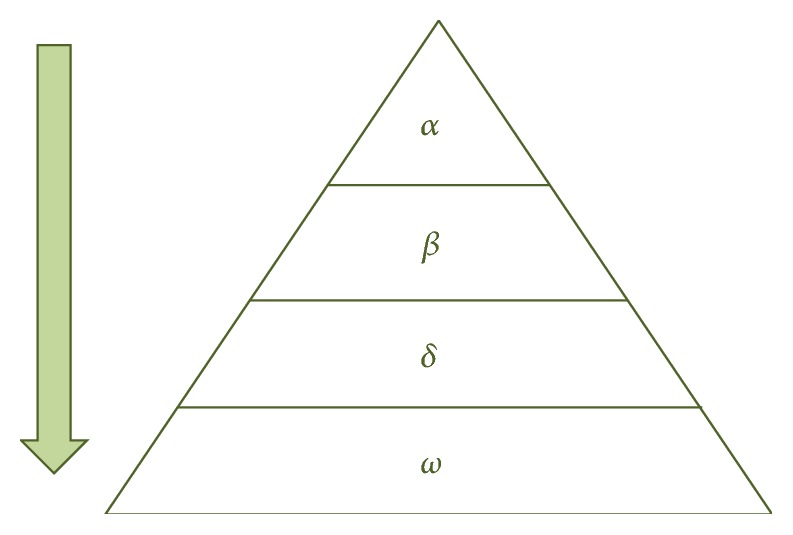
Hierarchy of grey wolf.

This algorithm is based on the hunting behavior of grey wolf and was proposed in [27]. A group of wolves has been between 5 and 12 wolves, and each wolf pack has a dominant hierarchy where the leaders are called alphas, and this type of wolves makes the most important decisions of the pack. The complete social dominant hierarchy is illustrated in Figure 2.

This algorithm is based on 5 points: social hierarchy, encircling prey, hunting, attacking prey, and search for prey. These points are explained as follows.

*Social Hierarchy*. The best solution is alpha (*α*), the second best solution is beta (*β*), the third best solution is delta (*δ*), and the rest of the population are considered as omega (*ω*), where the omega solutions follow alpha, beta, and delta wolves.

*Encircling Prey*. During the hunt process grey wolves encircle their prey. Mathematically model encircling behavior can be represented using the equations

(1) D→=C→·Xp→t−X→t,X→t+1=Xp→t−A→·D→,

where $\vec{A}$ and $\vec{C}$ are coefficient vectors, $\vec{X_{p}}$ is the prey position vector, $\vec{X}$ is the position vector of a grey wolf, and *t* is the current iteration. Vectors $\vec{A}$ and $\vec{C}$ are calculate by

(2) A→=2a→·r1→−a→,C→=2·r2→,

where $\vec{r_{1}}$ and $\vec{r_{2}}$ are random vectors with values in 0 and 1 and $\vec{a}$ is a vector with components that linearly decreased from 2 to 0 during iterations.

*Hunting*. It is assumed that alpha, beta, and delta are the best solutions; therefore, they have knowledge about location of prey, as these solutions are saved; the position of the other search agents is updated according to the position of the best search agent. This part is mathematically represented by

(3) Dα→=C1→·Xα→−X→,Dβ→=C2→·Xβ→−X→,Dδ→=C3→·Xδ→−X→,X1→=Xα→−A1→·Dα→,X2→=Xβ→−A2→·Dβ→,X3→=Xδ→−A3→·Dδ→,X→t+1=X1→+X2→+X3→3.

*Attacking Prey (Exploitation)*. $\vec{a}$ decreases from 2 to 0 during iterations and $\vec{A}$ has random numbers in an interval [−*a*, *a*] so the next position of a search agent will be any position between its current position and the prey.

*Search for Prey (Exploration)*. There are different components that allow having divergence and a good exploration. The divergence is mathematically modeled using $\vec{A}$, this part obliges solutions to diverge and to have a global search; meanwhile $\vec{C}$ contains values in an interval [0,2] and provides to the prey random weights to favor exploration and avoid a local optima problem. In Pseudocode 1, the pseudo code of the grey wolf optimizer is shown.

#### 2.1.2. Description of the Grey Wolf Optimizer for MGNN

The grey wolf optimizer seeks to optimize modular granular neural networks architectures. The optimized parameters are as follows:

1. Number of subgranules (modules).
2. Percentage of data for the training phase.
3. Learning algorithm (backpropagation algorithm for training the MGNN).
4. Goal error.
5. Number of hidden layers.
6. Number of neurons of each hidden layer.

**Figure 3:**
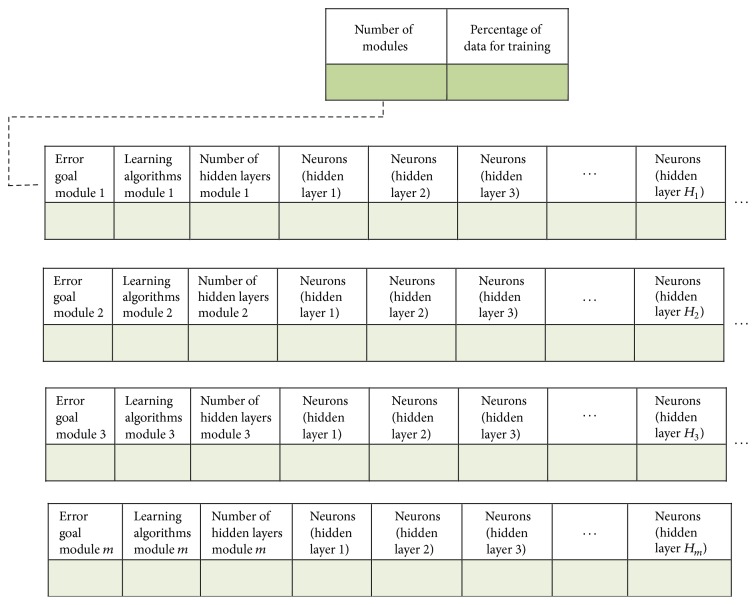
Structure of each search agent.

Each parameter is represented by a dimension in each solution (search agent), and to determine the total number of dimensions for each solution the next equation is used:

(4) $$\begin{array}{c} \text{Dimensions}=2+\left(\;3*m\;\right)+\left(\;m*h\;\right), \end{array}$$

where *m* is the maximum number of subgranules that the grey wolf optimizer can use and *h* is maximum of number of hidden layers per module that the optimizer can use to perform the optimization. The variables mentioned above can be established depending of the application or the database, and the values used for this work are mentioned in the next section. In Figure 3, the structure of each search agent is shown.

This optimizer aims to minimize the recognition error and the objective function is given by the equation:

(5) $$\begin{array}{c} f=\overset{m}{\underset{i=1}{\sum }}\left(\;\frac{\left(\sum_{j=1}^{n_{m}}X_{j}\right)}{n_{m}}\;\right), \end{array}$$

where *m* is the total number of subgranules (modules), *X*_*j*_ is 0 if the module provides the correct result and 1 if not, and *n*_*m*_ is total number of data/images used for testing phase in the corresponding module.

### 2.2. Proposed Method Applied to Human Recognition

One of the most important parameters of the architecture is its learning algorithm, backpropagation algorithms are used in the training phase to perform the learning, and 3 variations of this algorithm can be selected by the proposed optimizer: gradient descent with scaled conjugate gradient (SCG), gradient descent with adaptive learning and momentum (GDX), and gradient descent with adaptive learning (GDA). These 3 algorithms were selected because they have between demonstrated to be the fastest algorithms and with them better performances and results have been obtained [6, 7, 37–39].

The main comparisons with the proposed method are the optimizations proposed in [7, 37, 38]. In the first one a hierarchical genetic algorithm is developed, in the second and third work a firefly algorithm is developed to perform the MGNN optimization, and to have a fair comparison the number of individuals/fireflies and number of generations/iterations used in [7, 37, 38] are the same used by the proposed method in this work; obviously for the GWO these values are number of search agents and iterations. In Table 1, the values of the parameters used for each optimization algorithm are presented.

**Figure 4:**
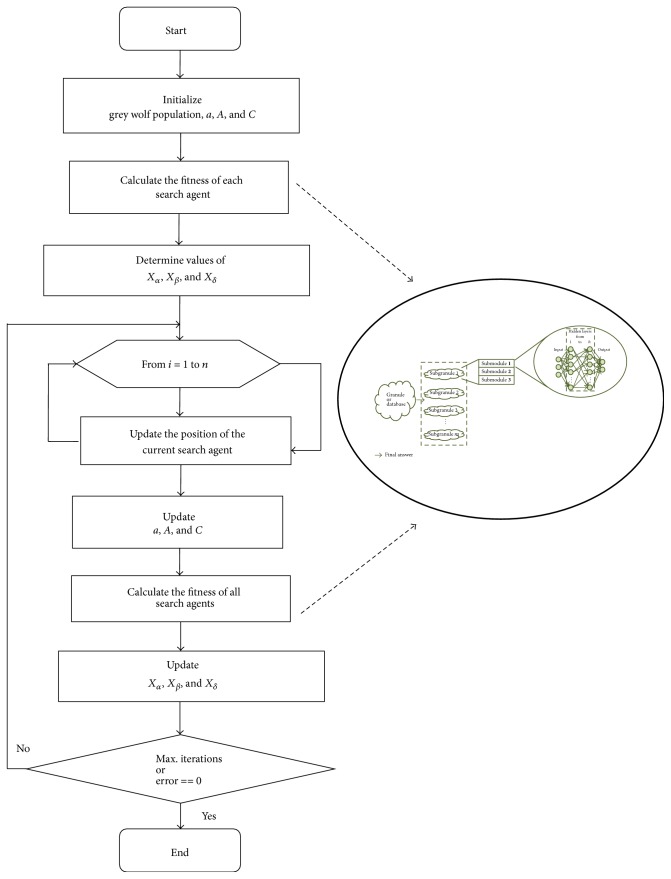
Diagram of the proposed method.

As it was mentioned, the number of dimensions is established using (4), where values such as *h* and *m* are established depending on the application. For this work as in [7, 37, 38], the minimum and maximum values used for the search space of each optimizer are shown in Table 2. The optimization techniques also have two stopping conditions: when the maximum number of iterations/generations is achieved and when the best solution has error value equal to zero. In Figure 4, the diagram of the proposed method is shown.

### 2.3. Data Selection, Databases, and Preprocessing

The description of the databases, data selection for each phase (training and testing), and the applied preprocessing are presented below.

#### 2.3.1. Data Selection

To understand the data selection, it is important to mention that the MGNNs as the MNNs and the conventional ANNs have two important phases:

- *First phase*: neural network learns information or patterns.
- *Second phase*: neural network simulates other pieces of information not given for learning.

**Figure 5:**
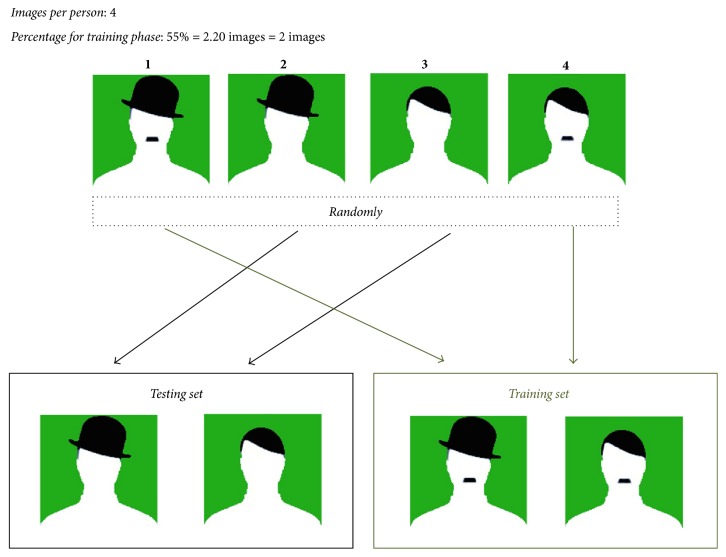
Example of selection of data for training and testing phase.

As it was observed, data selection is an important part of the neural network and for this reason in [7], a new method to select information or images was proposed. In the proposed data selection, depending of a percentage of data (a value between 20% and 80%) for the training phase, this percentage is converted to a number of images (depending of the number of images per person in the database), and randomly images for each phase are selected. In Figure 5, an example is illustrated, when a person has 4 images (as ear database) and 2 of them are for training phase.

#### 2.3.2. Database of Ear

**Figure 6:**
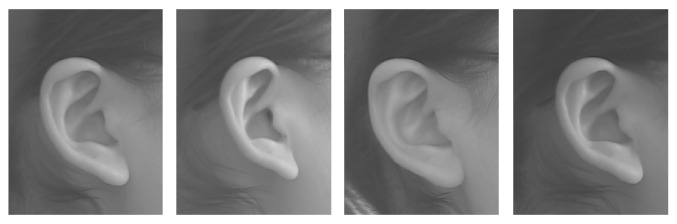
Sample of the Ear Recognition Laboratory database from the University of Science & Technology Beijing (USTB).

The ear database is from the Ear Recognition Laboratory of the University of Science & Technology Beijing (USTB). The database contains 77 persons, where each person has 4 images of one ear. The image dimensions are 300 × 400, with BMP format [47]. A sample of the images is shown in Figure 6.

#### 2.3.3. Database of Face (ORL)

**Figure 7:**
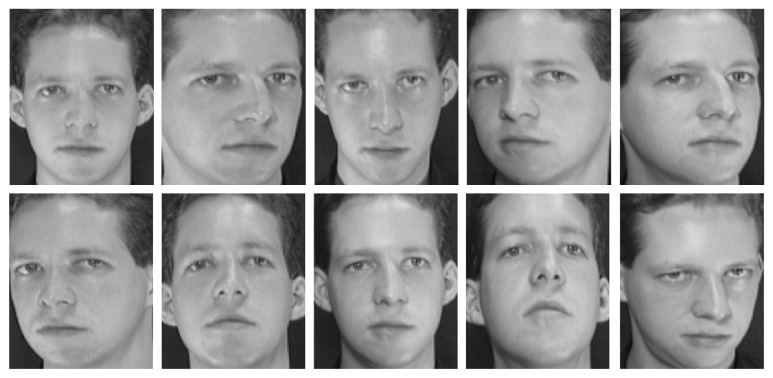
Sample of the ORL database from the AT&T Laboratories Cambridge.

The ORL database contains 40 persons, and each person has 10 images. This database is from the AT&T Laboratories Cambridge, where each image has a dimension of 92 × 112 pixels, with PGM format.  Figure 7 shows a sample of the images of this database [48].

#### 2.3.4. Database of Face (FERET)

**Figure 8:**
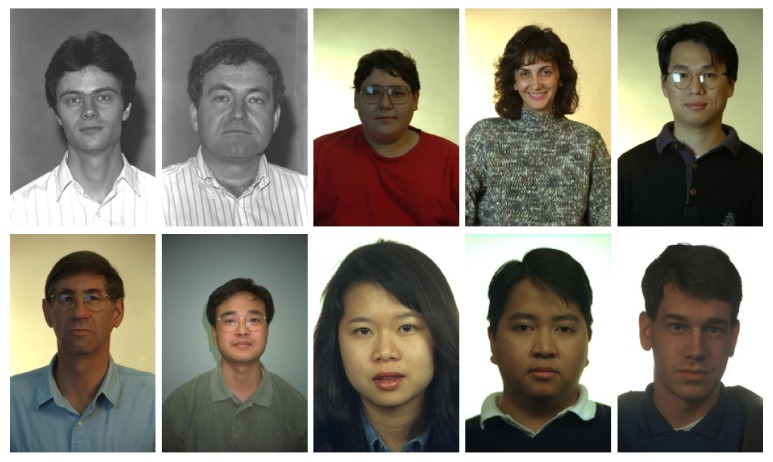
Sample of the FERET database.

The FERET database [49] contains 11338 images from 994 persons, and each image has a dimension of 512 × 768, pixels, with PGM format.  Figure 8 shows a sample of the images of this database.

#### 2.3.5. Database of Iris

**Figure 9:**
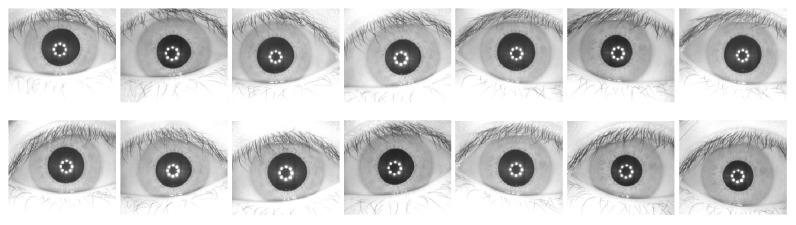
Sample of the iris database.

The iris database [50] contains 77 persons, each person has 14 images. The image dimensions are 320 × 280 pixels, with JPEG format.  Figure 9 shows a sample of the images of this database.

#### 2.3.6. Preprocessing

**Figure 10:**
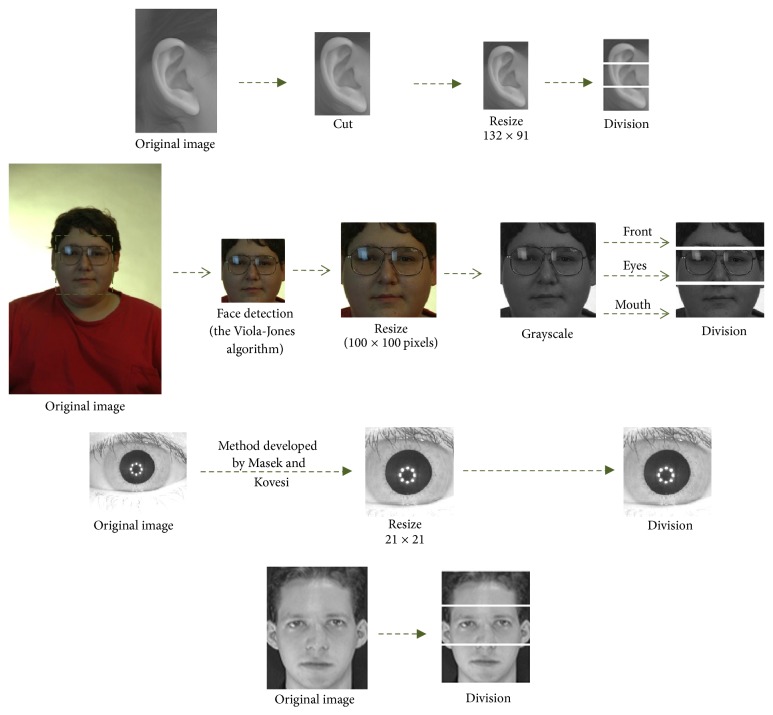
Sample preprocessing for the databases.

The preprocessing applied to these databases is simple because the focus of the proposed method is the optimization of the granulation. For the ear database, the ear image is manually cut, a resizing of the new image to 132 × 91 pixels is performed, and automatically the image is divided into three regions of interest (helix, shell, and lobe); this preprocessing was already performed in [7]. For the FERET database, the Viola-Jones algorithm [51, 52] was used to detect the face in each image, and a resizing of 100 × 100 pixels is performed to each image, converted to grayscale, and automatically the image is divided into three regions (front, eyes, and mouth). For iris database the method developed by Masek and Kovesi [53] is used to obtain the coordinates and radius of the iris and pupil to perform a cut in the iris, a resizing of 21 × 21 pixels is performed to each image, and finally, each image is automatically divided into three parts. For the ORL database, each image is automatically divided into three regions of interest (front, eyes, and mouth). The preprocessing process for these databases is shown in Figure 10.

## 3. Experimental Results

The proposed method is applied to human recognition and the results achieved are shown in this section. The main comparisons of the proposed method are against a hierarchical genetic algorithm proposed in [7] and a firefly algorithm proposed in [37, 38], where in [7, 38] the ear database is used; meanwhile in [37] the iris database is used and architectures of MGNNs are optimized. In [7, 38], two optimized tests for the MGNNs were performed, these tests in this work are replicated (30 trials/runs for each test), and to summarize only the 5 best results are shown. In [37], two optimized tests for the MGNNs were performed, the second test in this work is replicated (20 trials/runs), and to summarize also only the 5 best results are shown. For the ORL and FERET databases, 5 and 4 trials/runs were, respectively, performed to compare with other works.

### 3.1. Ear Results

The results achieved, using the ear database, are presented in this section. Each test is described as follows:

- *Test #1*: the search space for the percentage of data for the training phase is limited up to 80%; that is, the optimization technique can select up to this percentage of images of the total number of images per person.
- *Test #2*: in this test the search space for the percentage of data for the training phase is limited up to 50%.

#### 3.1.1. Test #1 Results for the Ear

In this test, the proposed grey wolf optimizer can use up to 80% of data for the training phase to design the MGNN architectures. In Table 3, the best 5 results using the proposed method in this work are shown.

**Figure 11:**
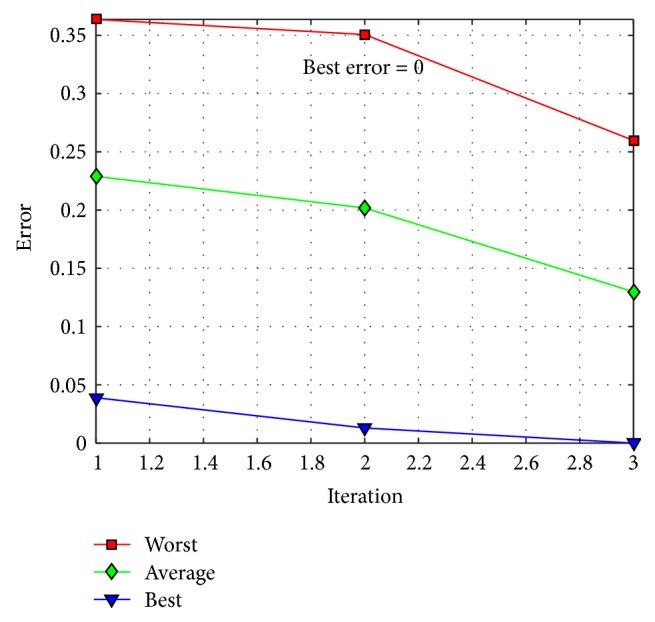
Convergence of trial #4.

**Figure 12:**
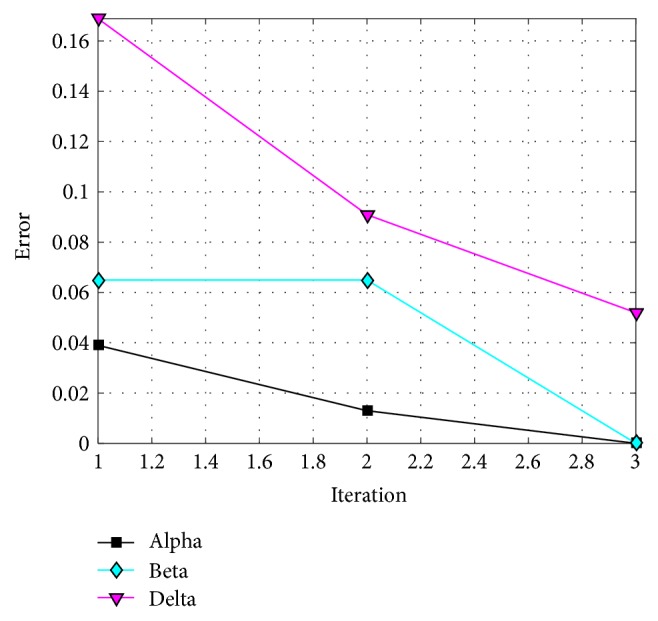
Alpha, beta, and delta behavior of trial #4.

The behavior of trial #4 is shown in Figure 11, where the best, the average, and the worst results of each iteration are shown. In Figure 12, alpha (first best solution), beta (second best solution), and delta (third best solution) behavior of trial #4 are shown. This trial was one of the fastest trials to obtain an error value equal to zero.

**Figure 13:**
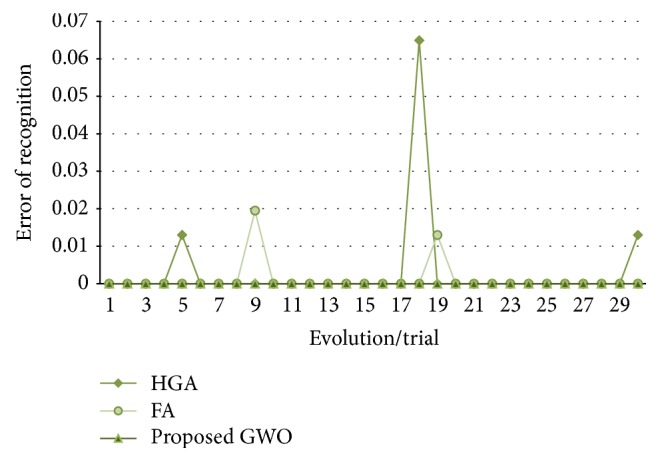
Obtained errors of recognition (up to 80%, ear).

In Figure 13, the recognition errors obtained by the proposed grey wolf optimizer, the HGA proposed in [7], and the FA proposed in [38] are shown.

In all the trials performed by the grey wolf optimizer an error equal to zero is obtained. In Table 4, a comparison of results between the proposed method and the work in [7, 38] is shown.

**Figure 14:**
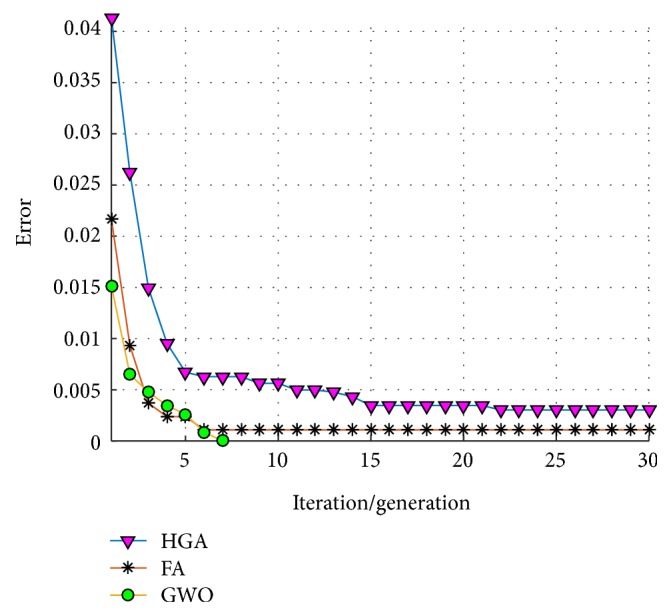
Average of convergence (test #1, ear).

An average of convergence of the 30 trials/runs of each optimization technique is shown in Figure 14, where it can be observed that the GWO always found an error equal to zero in the first 5 iterations; meanwhile the HGA and the FA in some runs did not obtain this value.

#### 3.1.2. Test #2 Results for Ear

In this test, the proposed grey wolf optimizer can use up to 50% of data for the training phase to design the MGNNs architectures. In Table 5, five architectures with the best results are shown.

**Figure 15:**
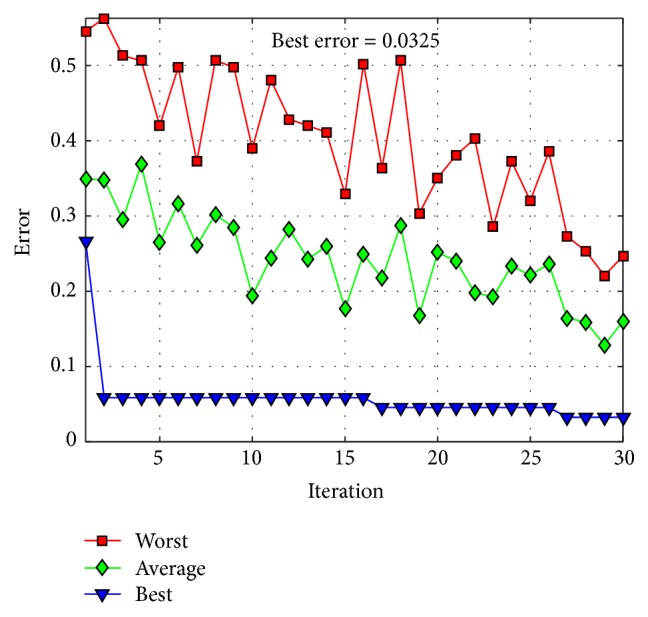
Convergence of trial #2.

**Figure 16:**
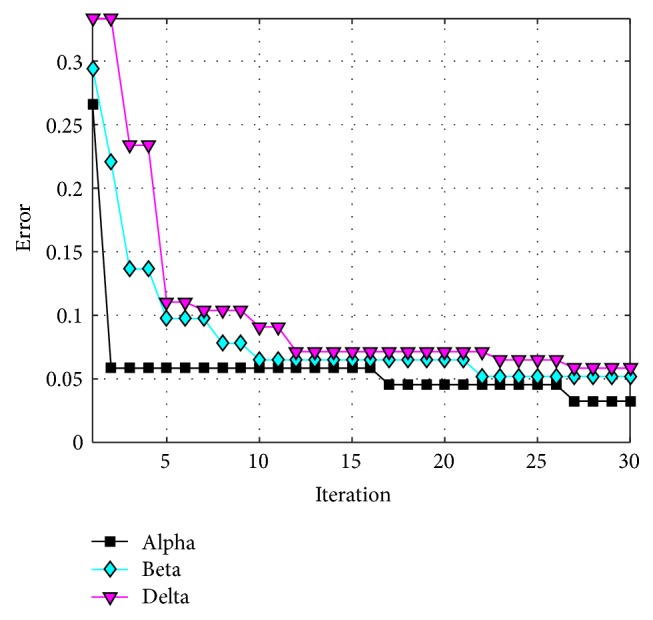
Convergence of trial #2.

The behavior of trial #2 is shown in Figure 15, where the best, the average, and the worst results of each iteration are shown. In Figure 16, the alpha (first best solution), beta (second best solution), and delta (third best solution) behaviors of trial #2 are shown. This trial was one of the best trials, where an error of recognition equal to 0.325 is obtained.

**Figure 17:**
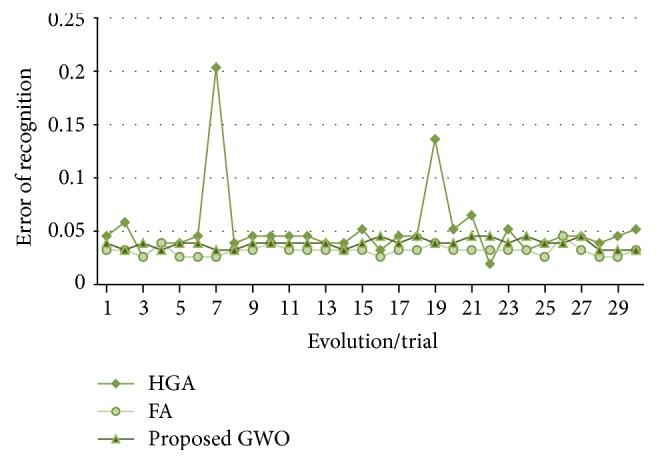
Obtained errors of recognition (up to 50%, ear).

In Figure 17, the errors of recognition obtained by the proposed grey wolf optimizer, the HGA proposed in [7] and the FA proposed in [38] for test #2, are shown. It can be visually seen that the results obtained by grey wolf optimizer and firefly algorithm are more stable than the HGA.

In Table 6, a comparison of results between the proposed method and [7, 38] is shown. The best result is obtained by the HGA, but the average is slightly improved by the firefly algorithm; meanwhile the worst errors are improved by the proposed method and the firefly algorithm.

**Figure 18:**
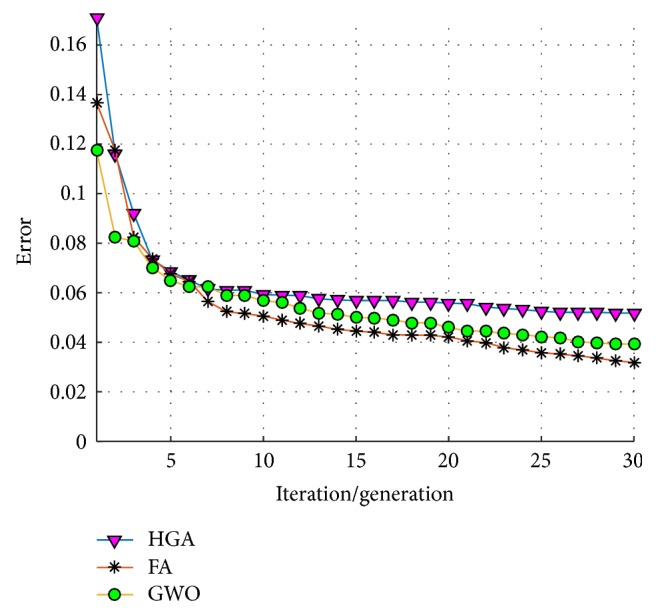
Average of convergence (test #2, ear).

An average of convergence of the 30 trials/runs of each optimization technique is shown in Figure 18, where the HGA tends in a general behavior to stagnate more than the GWO and the FA.

### 3.2. Face Results (ORL)

The results achieved, using the ORL database, are presented in this section. For this database 2 tests were also performed, but to compare with other works the percentage of data for the training phase is set fixed. Each test is described as follows:

- *Test #1*: the percentage of data for the training phase is set to 80%.
- *Test #2*: the percentage of data for the training phase is set to 50%.

#### 3.2.1. Test #1 Results for Face

In this test, the proposed grey wolf optimizer uses 80% of data for the training phase to design the MGNNs architectures. In Table 7, five architectures with the best results are shown.

**Figure 19:**
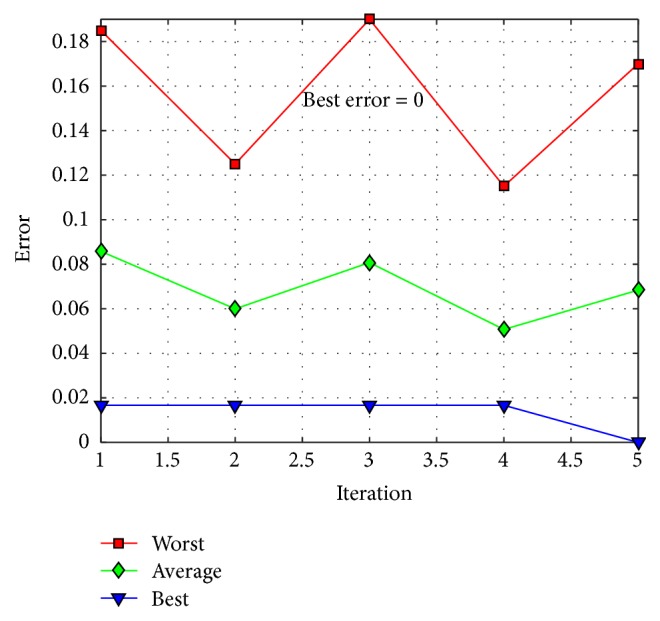
Convergence of trial #5.

**Figure 20:**
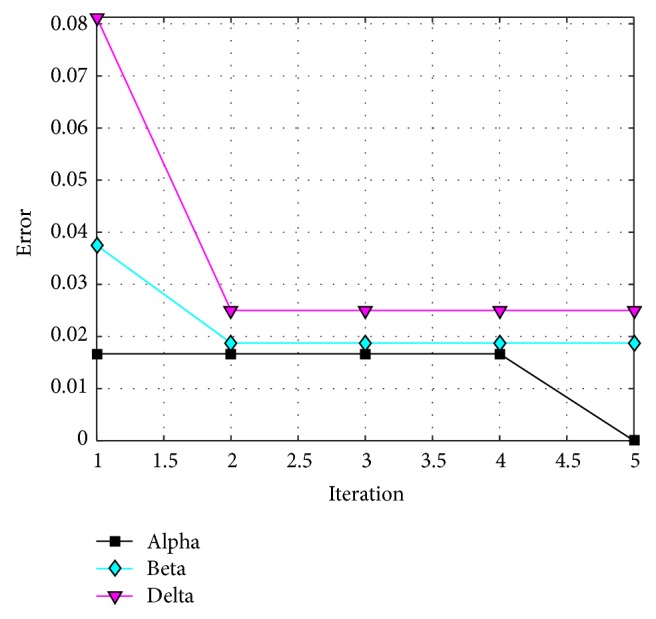
Convergence of trial #5.

The behavior of trial #5 is shown in Figure 19, where the best, the average, and the worst results of each iteration are shown. In Figure 20, the alpha (first best solution), beta (second best solution), and delta (third best solution) behaviors of trial #5 are shown. This trial was one of the fastest trials to obtain an error value equal to zero.

**Figure 21:**
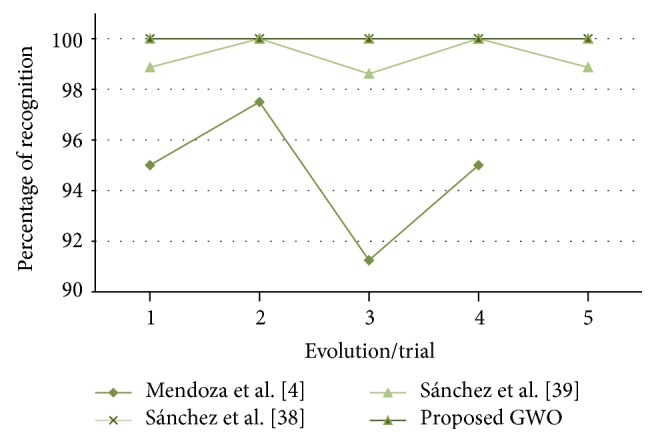
Obtained recognition rates (test #1, ORL database, comparison 1).

In Figure 21, the recognition rates obtained by [4, 38, 39] and the proposed grey wolf optimizer are shown. The proposed method and the firefly proposed in [38] allow obtaining a recognition rate of 100%.

In Table 8, a comparison of results is presented. The best result is obtained by the work in [38, 39] and the proposed method, but the average and the worst error are improved by the proposed method and the firefly algorithm.

#### 3.2.2. Test #2 Results for Face

In this test, the proposed grey wolf optimizer uses 50% of data for the training phase to design the MGNNs architectures. In Table 9, the best 5 results using the proposed method in this work are shown.

**Figure 22:**
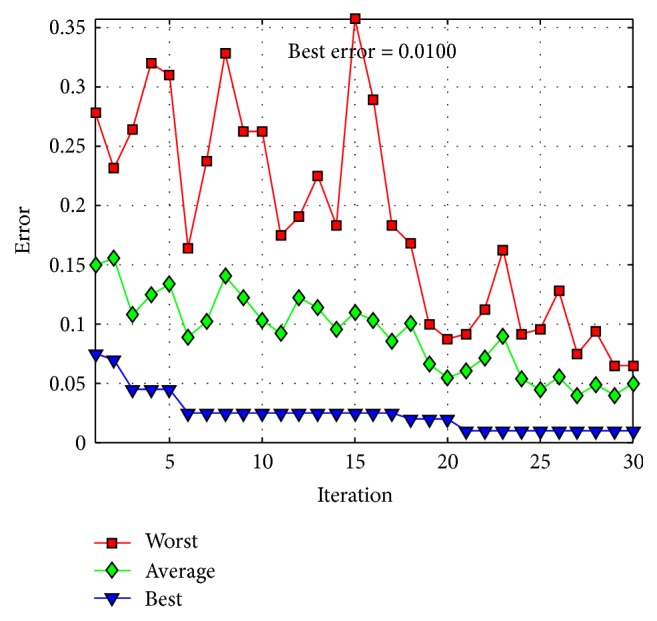
Convergence of trial #1.

**Figure 23:**
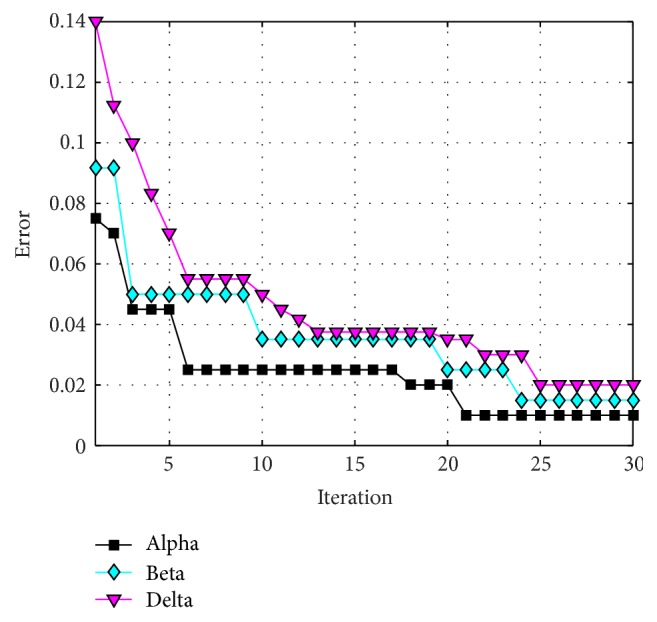
Convergence of trial #1.

The behavior of trial #1 is shown in Figure 22, where the best, the average, and the worst results of each iteration are shown. In Figure 23, the alpha (first best solution), beta (second best solution), and delta (third best solution) behaviors of trial #1 are shown. This trial was one of the best trials, where an error of recognition equal to 0.0100 is obtained.

**Figure 24:**
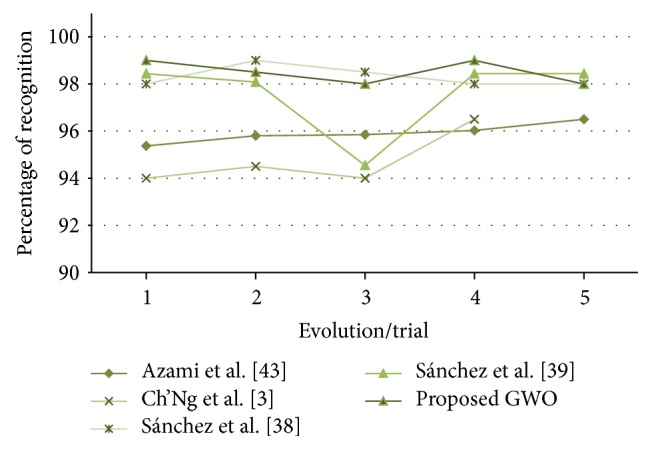
Obtained recognition rates (test #2, ORL database, comparison 2).

In Figure 24, the recognition rates obtained by [3, 38, 39, 43] and the proposed method are shown.

In Table 10, a comparison of results between the proposed method and the other works is shown. The best and the worst error are improved by the proposed method and the firefly algorithm, but the average of recognition is slightly improved by the proposed method.

### 3.3. Iris Results

In this test, the proposed grey wolf optimizer uses up to 80% of data for the training phase to design the MGNNs architectures as in [37, 44]. In Table 11, five architectures with the best results are shown.

**Figure 25:**
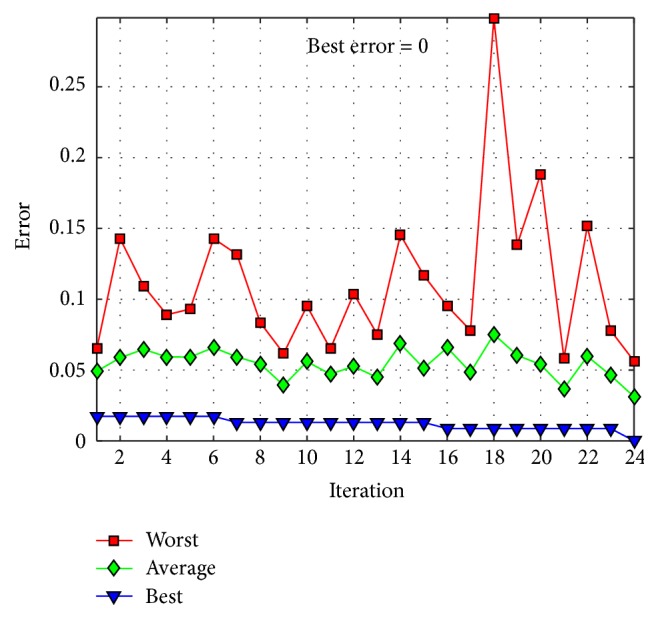
Convergence of trial #2.

**Figure 26:**
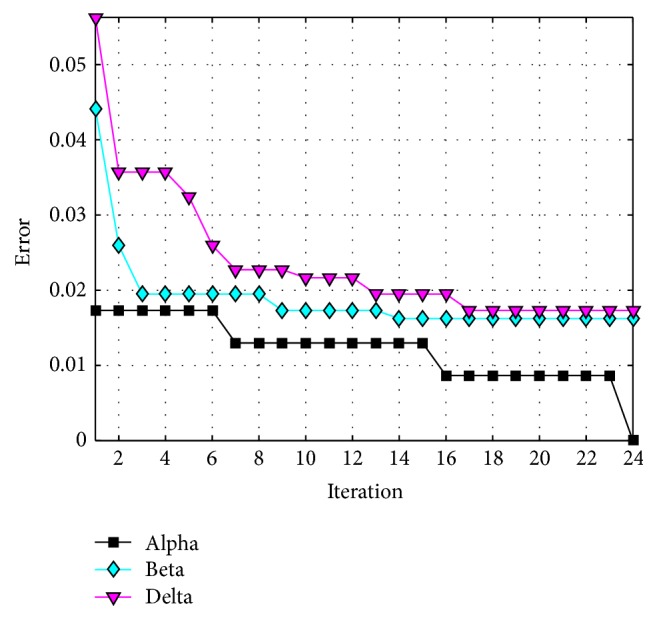
Convergence of trial #2.

The behavior of trial #2 is shown in Figure 25, where the best, the average, and the worst results of each iteration are shown. In Figure 26, the alpha (first best solution), beta (second best solution), and delta (third best solution) behaviors of trial #2 are shown. This trial was one of the best trials, where an error of recognition equal to 0 is obtained.

**Figure 27:**
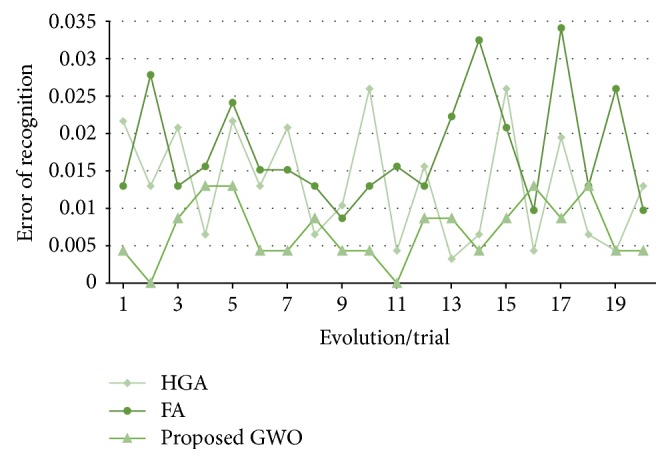
Obtained recognition rates (iris database).

In Figure 27, the errors of recognition obtained by [37, 44] and the proposed method are presented.

In Table 12, a comparison of results is presented. The best, the average, and the worst errors are improved by the proposed method.

**Figure 28:**
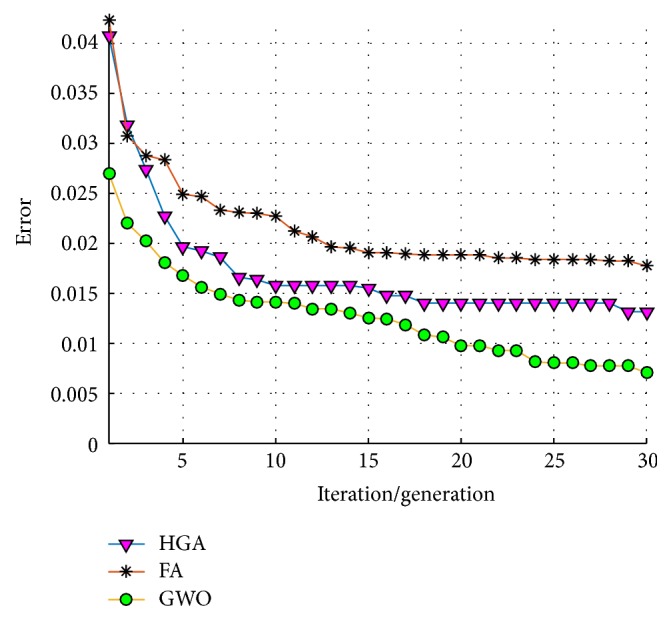
Average of convergence (iris).

An average of convergence of the 20 trials/runs of each optimization technique is shown in Figure 28, where although these techniques does not tend to stagnate for a long time, the GWO tends to convergence faster with better results.

### 3.4. Summary Results

**Figure 29:**
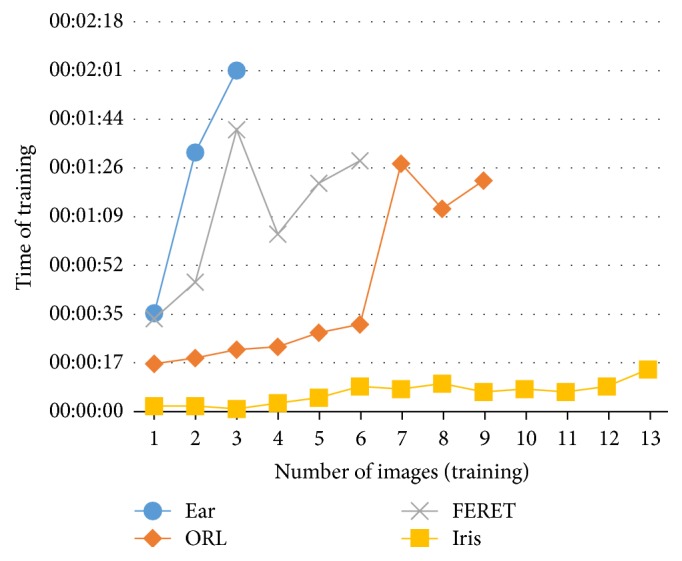
Average of training time.

**Figure 30:**
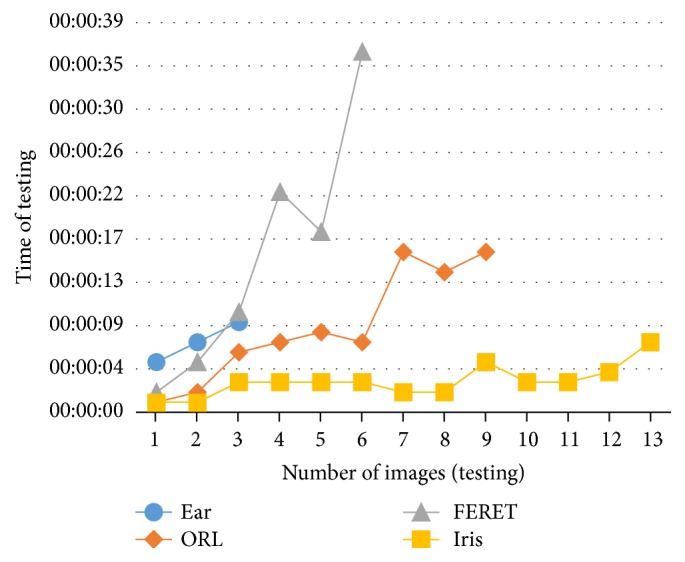
Average of training time.

Summary of results and comparison with other works using the same databases and neural networks are shown in this section. The testing time of a set of images depends on the number of images and their size, but the training time also depends on the neural network architecture (number of hidden layers, neurons in each hidden layers, and number of modules) and learning factors (initial weights and error goal, among others). An approximation of the training and testing times for each search agent is, respectively, shown in Figures 29 and 30.

In Table 13 a summary of each database setup is shown. It can be noticed that the Iris database has more images in each test, but images size is smaller than the other databases; for this reason the training and testing times for this database are the smallest ones. In the case of ear database the number of images is smaller than the other databases but the size of its images is bigger, so the training and testing times tend to increase.

In Table 14, the summary of results obtained using the GWO applied to the ear, face, and iris database is shown.

In [7], modular granular neural networks are proposed and are compared with conventional neural networks using a hierarchical genetic algorithm to design neural networks architectures. In [38], the design of modular granular neural networks architectures is proposed using a firefly algorithm. In [45], the architectures of modular neural networks are designed using a hierarchical genetic algorithm but without a granular approach; that is, the number of modules and the number of persons learned by each modules always were left fixed. In Table 15, the comparisons among the optimized results obtained using the proposed method and other optimized works are presented, where the average was improved for the ear database by the proposed method (test #1, using 3 images) and the firefly algorithm (test #2, using 2 images).

In Table 16, the 4-fold cross-validation results for the ear database are shown, where for each training set 3 images for each person were used.

In [43], a neural network is proposed based on a conjugate gradient algorithm (CGA) and a principal component analysis. In [3], the principal components analysis (PCA) and a linear discriminant analysis (LDA) are used. In [38], a firefly algorithm is developed to design modular granular neural networks architectures. In [39], modular neural network with a granular approach is used, but in that work, the granulation is performed using nonoptimized training to assign a complexity level to each person and to form subgranules with persons that have the same complexity level. That method was recommended for databases with a large numbers of people. In [4], a comparison of fuzzy edge detectors based on the image recognition rate as performance index calculated with neural networks is proposed. In Table 17, the comparisons among the optimized results obtained using the proposed method and other optimized works for the face database are presented, where the best, average, and worst values were improved for this database by the proposed method and the firefly algorithm for test #1 (using 8 images) and in test #2 (using 5 images); the average is only improved by the proposed method.

In Table 18, the 5-fold cross-validation results are shown, where for each training set 4 images for each person were used.

In [46] a scale invariant feature transform (SIFT) is proposed. In Table 19, the comparisons among the results obtained using the proposed method and the other work for the FERET database are presented.

In Table 20, the 5-fold cross-validation results are shown, where for each training set 4 images for each person were used.

In [44] and [37], a hierarchical genetic algorithm and a firefly algorithm are, respectively, proposed to optimize modular granular neural networks using iris as biometric measure. The main difference between these works is that in the first and the second one there is no a subdivision of each image as in the proposed method where submodules are experts in parts of the image. In Table 21, the comparison between the optimized results obtained using the proposed method and the other optimized works is presented.

In Table 22, the 5-fold cross-validation results are shown, where for each training set 11 images for each person were used.

## 4. Statistical Comparison of Results

The results obtained by the proposed method are visually better than the other works; now statistical *t*-tests are performed in order to verify if there is enough evidence to say that the results of the proposed method are better. In these *t*-tests, the recognition rates and errors previously presented were used.

### 4.1. Statistical Comparison for Test #1

In Table 23, the values obtained in the *t*-test between [7] and [38] and the proposed method are shown, where the *t*-values were, respectively, 1.38 and 1.41; this means that there is no sufficient evidence to say that ear results for test #1 were improved with the proposed method.

**Figure 31:**
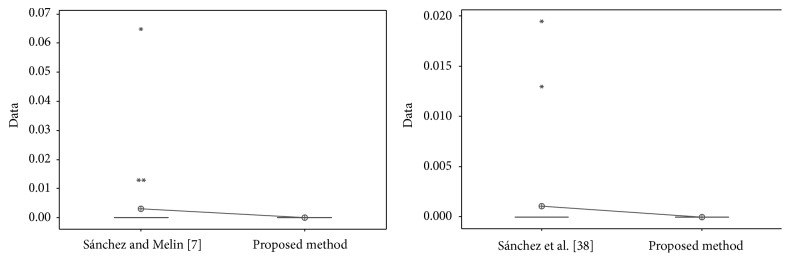
Sample distribution (test #1, ear database).

In Figure 31, the distribution of the samples is shown, where it can be observed that the samples are very close to each other.

**Figure 32:**
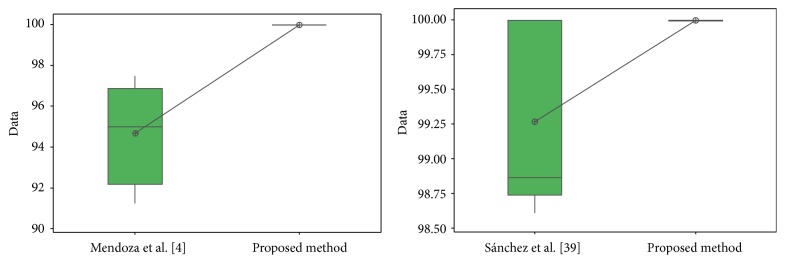
Sample distribution (test #1, ORL database).

For the ORL database in test #1, the different values obtained in the *t*-test between the proposed method and [4, 39] are shown in Table 24. The *t*-values were 4.12 and 2.42; this means that there is sufficient evidence to say that the results were improved using the proposed method. In Figure 32, the distribution of the samples is shown. It can be observed that samples of [39] are very separated from each other.

**Figure 33:**
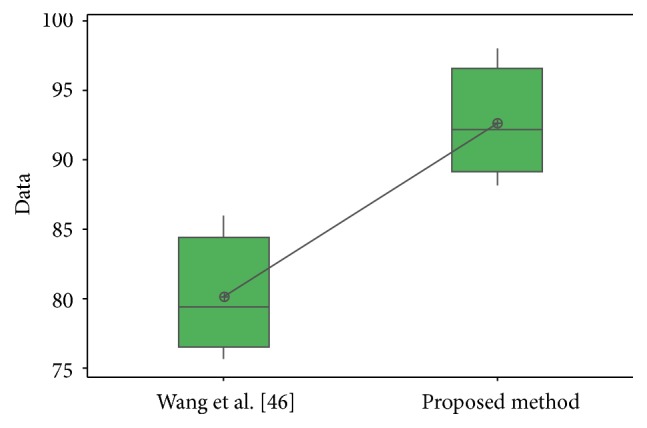
Sample distribution (FERET database).

For the FERET database, the different values obtained in the *t*-test between the proposed method and [46] are shown in Table 25. The *t*-value was 4.24; this means that there is sufficient evidence to say that the results were improved using the proposed method. In Figure 33, the distribution of the samples is shown.

For the iris database, the different values obtained in the *t*-test between the proposed method and [44] and [37] are shown in Table 26. The *t*-values were, respectively, 3.18 and 5.62; this means that there is sufficient evidence to say that the results were improved using the proposed method.

**Figure 34:**
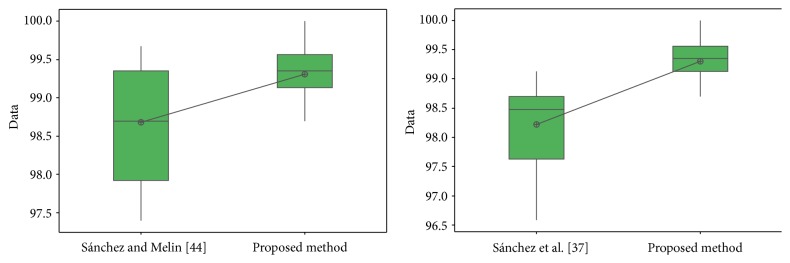
Sample distribution (iris database).

In Figure 34, the distribution of the samples is shown. It can be observed that samples of [44] are more separated from each other than in [37].

### 4.2. Statistical Comparison for Test #2

In Table 27, the values obtained in the *t*-test between [7] and [38] and the proposed method for ear database are shown, where the *t*-values were, respectively, 2.09 and −5.70; this means that there is sufficient evidence to say that face results were improved with the proposed method only versus [7].

**Figure 35:**
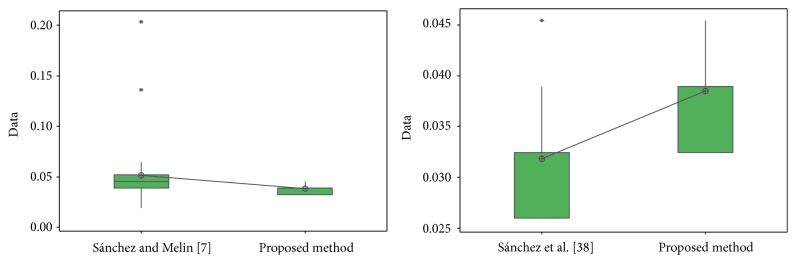
Sample distribution (test #2, ear database).

In Figure 35, the distribution of the samples is shown, where it can be observed that the samples for [7] and the proposed method are closer than the proposed method and [38]. The distribution of the proposed method and [38] seems to be uniform.

The different values obtained in the *t*-test for the face database between the proposed method and [43], [3], [38], and [39] are shown in Table 28. The *t*-values were, respectively, 8.96, 5.90, 0.67, and 1.15; this means that only compared with [3, 43] there is sufficient evidence to say that the face results were improved using the proposed method.

**Figure 36:**
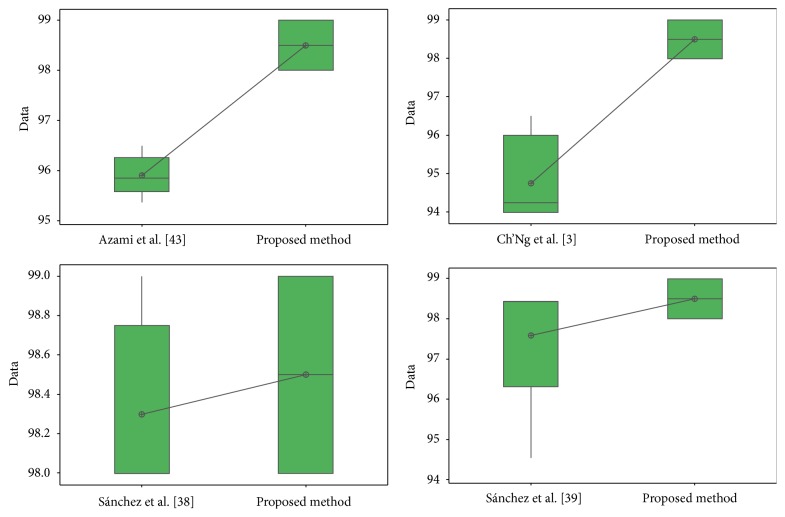
Sample database (test #2, ORL database).

In Figure 36, the distribution of the samples is shown, where it can be observed that the samples are very close between the proposed method and [38, 39].

## 5. Conclusions

In this paper, the design of modular granular neural network architectures using a grey wolf optimizer is proposed. The design of these architectures consists in the number of modules, percentage of data for the training phase, error goal, learning algorithm, number of hidden layers, and their respective number of neurons. As objective function this optimizer seeks to minimize the recognition error applying the proposed method to human recognition, where benchmark databases of ear and face biometric measures were used to prove the effectiveness of the proposed method. Statistical comparisons were performed to know if there is sufficient evidence of improvements using the proposed method, mainly with previous works, where a hierarchical genetic algorithm and a firefly algorithm were developed and also use MGNNs, but more comparisons with other works were also performed. As a conclusion, the proposed method has been shown which improves recognition rates in most of the comparisons, especially when the granular approach is not used. An improvement provided by the grey wolf optimizer over the genetic algorithm and the firefly algorithm lies in the fact that the first one allows having the first three best solutions (alpha, beta, and delta) and their others search agents will update their position based on them; otherwise, the genetic algorithm only has a best solution in each iteration, and the firefly algorithm updates the position of the fireflies by evaluating couples of fireflies, where if one firefly is not better than the other their move will be random. This allows the GWO to have greater stability in its trials and in its results. It is important to mention that the results shown in this work were performed using different databases; this prove that the proposed method was designed to be easily adaptable depending of the number of persons and the number of images independently of the biometric measure used. In future works, the proposed method will seek to reduce the complexity of the MGNNs architectures and to minimize the percentage of information and subgranules to design MGNNs.

## Figures and Tables

**Pseudocode 1:**
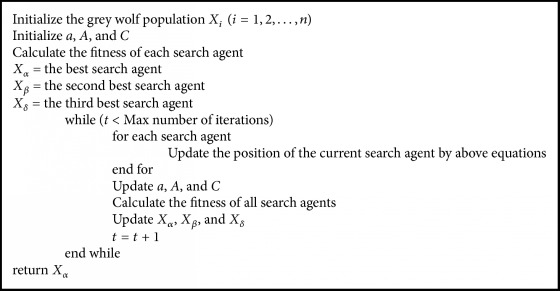
Pseudocode of the grey wolf optimizer.

**Table 1:** Table of parameters.

HGA [7]		FA [37, 38]		GWO	
Parameter	Value	Parameter	Value	Parameter	Value
Individuals (*n*)	10	Fireflies	10	Search agents (*n*)	10
Maximum number of generations (*t*)	30	Maximum number of iterations (*t*)	30	Maximum number of iterations (*t*)	30

**Table 2:** Table of values for search space.

Parameters of MNNs	Minimum	Maximum
Modules (*m*)	1	10
Percentage of data for training	20	80
Error goal	0.000001	0.001
Learning algorithm	1	3
Hidden layers (*h*)	1	10
Neurons for each hidden layers	20	400

**Table 3:** The best 10 results (test #1, ear).

Trial	Images		Number of hidden layers and number of neurons	Persons per module	Rec. rate	Error
	Training	Testing				
1	80% (1, 2, and 3)	20% (4)	5 (126, 96, 179, 239, 37) 4 (188, 196, 93, 171) 5 (109, 107, 110, 168, 29)	Module #1 (1 to 12) Module #2 (13 to 40) Module #3 (41 to 77)	100% (77/77)	0

2	69% (2, 3 and 4)	31% (1)	5 (222, 238, 113, 27, 75) 4 (151, 53, 99, 79) 2 (209, 31) 2 (144, 71) 4 (30, 218, 194, 199) 4 (25, 81, 239, 20) 5 (237, 43, 83, 102, 128)	Module #1 (1 to 5) Module #2 (6 to 21) Module #3 (22 to 31) Module #4 (32 to 46) Module #5 (47 to 63) Module #6 (64 to 73) Module #7 (74 to 77)	100% (77/77)	0

3	66% (2, 3, and 4)	34% (1)	5 (141, 70, 120, 158, 242) 4 (124, 55, 23, 243) 3 (96, 186, 213) 4 (28, 62, 51, 42) 1 (223)	Module #1 (1 to 34) Module #2 (35 to 40) Module #3 (41 to 44) Module #4 (45 to 75) Module #5 (76 to 77)	100% (77/77)	0

4	74% (2, 3, and 4)	26% (1)	5 (139, 97, 200, 121, 231) 5 (204, 114, 164, 216, 138) 5 (195, 137, 124, 71, 86) 5 (144, 70, 92, 220, 63) 5 (119, 176, 154, 167, 161) 4 (199, 162, 96, 65)	Module #1 (1 to 6) Module #2 (7 to 29) Module #3 (30 to 50) Module #4 (51 to 58) Module #5 (59 to 71) Module #6 (72 to 77)	100% (77/77)	0

5	63% (2, 3, and 4)	37% (1)	5 (136, 183, 149, 193, 161) 5 (181, 132, 175, 140, 155)	Module #1 (1 to 68) Module #2 (69 to 77)	100% (77/77)	0

**Table 4:** Comparison of results (test #1, ear).

Method	Best	Average	Worst
HGA [7]	100%	99.70%	93.50%
	0	0.00303	0.0649
FA [38]	100%	99.89%	98.05%
	0	0.0011	0.0195
Proposed GWO	100%	100%	100%
	0	0	0

**Table 5:** The best 10 results (test #2, ear).

Trial	Images		Number of hidden layers and number of neurons	Persons per module	Rec. rate	Error
	Training	Testing				
2	43% (2 and 3)	57% (1 and 4)	5 (115, 49, 187, 122, 194) 5 (182, 139, 50, 217, 54) 5 (132, 182, 56, 187, 159) 5 (167, 132, 121, 123, 219) 4 (116, 195, 54, 174) 5 (157, 108, 166, 95, 88) 5 (116, 119, 76, 121, 94) 5 (102, 58, 69, 111, 42)	Module #1 (1 to 9) Module #2 (10 to 22) Module #3 (23 to 33) Module #4 (34 to 36) Module #5 (37 to 51) Module #6 (52 to 63) Module #7 (64 to 75) Module #8 (76 to 77)	96.75% (149/154)	0.0325

4	48% (2 and 3)	52% (1 and 4)	4 (98, 136, 165, 141) 3 (176, 104, 215) 4 (142, 222, 65, 28) 5 (97, 139, 129, 99, 28) 4 (225, 83, 188, 34)	Module #1 (1 to 26) Module #2 (27 to 39) Module #3 (40 to 55) Module #4 (56 to 65) Module #5 (66 to 77)	96.75% (149/154)	0.0325

7	49% (2 and 3)	51% (1 and 4)	5 (201, 84, 169, 113, 131) 5 (199, 189, 62, 159, 151) 5 (104, 129, 88, 166, 66) 5 (123, 96, 52, 26, 67) 5 (125, 141, 86, 77, 105) 5 (121, 145, 87, 122, 31) 5 (36, 126, 146, 143, 145) 5 (126, 140, 88, 173, 206)	Module #1 (1 to 5) Module #2 (6 to 17) Module #3 (18 to 32) Module #4 (33 to 34) Module #5 (35 to 40) Module #6 (41 to 51) Module #7 (52 to 63) Module #8 (64 to 77)	96.75% (149/154)	0.0325

8	39% (2 and 3)	61% (1 and 4)	5 (125, 75, 69, 114, 140) 5 (138, 157, 101, 164, 98) 5 (76, 78, 86, 135, 70) 4 (74, 53, 57, 73) 5 (123, 55, 75, 125, 143) 5 (99, 118, 149, 224, 67) 5 (130, 184, 156, 180, 153)	Module #1 (1 to 11) Module #2 (12 to 14) Module #3 (15 to 27) Module #4 (28 to 33) Module #5 (34 to 43) Module #6 (44 to 57) Module #7 (58 to 77)	96.75% (149/154)	0.0325

14	40% (2 and 3)	60% (1 and 4)	5 (58, 26, 159, 123, 106) 5 (157, 156, 197, 22, 112) 4 (215, 78, 97, 220) 5 (120, 68, 219, 194, 58) 5 (142, 185, 141, 33, 187) 5 (108, 160, 61, 100, 54)	Module #1 (1 to 12) Module #2 (13 to 20) Module #3 (21 to 40) Module #4 (41 to 52) Module #5 (53 to 66) Module #6 (67 to 77)	96.75% (149/154)	0.0325

**Table 6:** Comparison of results (test #2, ear).

Method	Best	Average	Worst
HGA [7]	98.05%	94.82%	79.65%
	0.01948	0.0518	0.20346
FA [38]	97.40%	96.82%	95.45%
	0.0260	0.0318	0.04545
Proposed GWO	96.75%	96.15%	95.45%
	0.03247	0.03853	0.04545

**Table 7:** The results for face database (test #1, ORL).

Trial	Images		Number of hidden layers and number of neurons	Persons per module	Rec. rate	Error
	Training	Testing				
1	80% (1, 2, 3, 4, 7, 8, 9, and 10)	20% (5 and 6)	5 (109, 109, 69, 74, 210) 5 (175, 32, 170, 214, 86) 4 (117, 52, 134, 197) 4 (190, 162, 99, 81) 5 (111, 130, 247, 160, 64) 4 (111, 250, 116, 127)	Module #1 (1 to 4) Module #2 (5 to 12) Module #3 (13 to 15) Module #4 (16 to 24) Module #5 (25 to 33) Module #6 (34 to 40)	100% (80/80)	0

2	80% (1, 3, 4, 5, 6, 7, 8, and 10)	20% (2 and 9)	5 (52, 188, 138, 154, 71) 5 (216, 183, 74, 142, 112) 5 (73, 204, 139, 94, 114) 5 (101, 124, 144, 207, 133) 4 (96, 205, 157, 238) 5 (46, 160, 86, 119, 105) 5 (138, 169, 152, 146, 48) 5 (32, 65, 173, 156, 56)	Module #1 (1 to 5) Module #2 (6 to 15) Module #3 (16 to 17) Module #4 (18 to 19) Module #5 (20 to 29) Module #6 (30 to 32) Module #7 (33 to 38) Module #8 (39 to 40)	100% (80/80)	0

3	80% (1, 2, 4, 5, 7, 8, 9, and 10)	20% (3 and 6)	5 (158, 67, 80, 49, 124) 5 (138, 72, 51, 87, 218) 5 (138, 176, 108, 21, 139) 5 (136, 46, 66, 41, 68) 5 (182, 40, 246, 104, 45) 5 (126, 202, 171, 45, 228) 5 (228, 153, 133, 199, 85) 4 (98, 140, 72, 188)	Module #1 (1 to 3) Module #2 (4 to 5) Module #3 (6 to 13) Module #4 (14 to 18) Module #5 (19 to 23) Module #6 (24 to 25) Module #7 (26 to 30) Module #8 (31 to 40)	100% (80/80)	0

4	80% (1, 3, 4, 5, 7, 8, 9, and 10)	20% (2 and 6)	5 (39, 55, 21, 84, 210) 1 (224) 3 (98, 204, 243) 5 (61, 86, 237, 49) 2 (199, 62) 1 (180) 5 (206, 29, 240, 215, 105)	Module #1 (1 to 7) Module #2 (8 to 9) Module #3 (10 to 12) Module #4 (13 to 17) Module #5 (18 to 26) Module #6 (27 to 34) Module #7 (35 to 40)	100% (80/80)	0

5	80% (1, 2, 3, 5, 6, 7, 8, and 10)	20% (4 and 9)	5 (75, 156, 197, 128, 233) 5 (225, 87, 193, 58, 182) 5 (161, 240, 36, 157, 151) 5 (228, 222, 64, 102, 132) 5 (161, 50, 80, 175, 105) 5 (150, 105, 194, 122, 80) 5 (121, 116, 122, 88, 42) 5 (66, 210, 92, 48, 179)	Module #1 (1 to 4) Module #2 (5 to 13) Module #3 (14 to 16) Module #4 (17 to 23) Module #5 (24 to 26) Module #6 (27 to 29) Module #7 (30 to 31) Module #8 (32 to 40)	100% (80/80)	0

**Table 8:** Comparison of results (test #1, ORL).

Method	Best	Average	Worst
Mendoza et al. [4]	97.50%	94.69%	91.5%
Sánchez et al. [38]	100%	100%	100%
Sánchez et al. [39]	100%	99.27%	98.61%
Proposed GWO	100%	100%	100%

**Table 9:** The results for face database (test #2, ORL).

Trial	Images		Number of hidden layers and number of neurons	Persons per module	Rec. rate	Error
	Training	Testing				
1	50% (2, 3, 4, 7, and 9)	50% (1, 5, 6, 8 and, 10)	5 (139, 149, 64, 49, 69) 5 (112, 89, 137, 112, 203) 5 (109, 141, 115, 142, 206) 5 (69, 183, 84, 33, 233) 5 (43, 127, 176, 236, 39) 5 (124, 192, 92, 92, 193) 5 (70, 188, 227, 165, 98) 5 (75, 79, 128, 171, 159)	Module #1 (1 to 5) Module #2 (6 to 12) Module #3 (13 to 17) Module #4 (18 to 22) Module #5 (23 to 30) Module #6 (31 to 34) Module #7 (35 to 36) Module #8 (37 to 40)	99% (198/200)	0.0100

2	50% (1, 2, 4, 5, and 7)	50% (3, 6, 8, 9 and, 10)	5 (141, 99, 172, 88, 81) 4 (198, 101, 244, 148) 5 (159, 31, 175, 125, 168) 5 (31, 90, 125, 116, 111) 5 (102, 107, 110, 87, 21) 5 (113, 78, 55, 184, 209) 5 (248, 108, 150, 88, 40) 4 (119, 136, 90, 126) 3 (213, 71, 127) 4 (207, 131, 182, 48)	Module #1 (1 to 7) Module #2 (8 to 12) Module #3 (13 to 15) Module #4 (16 to 18) Module #5 (19 to 21) Module #6 (22 to 23) Module #7 (24 to 30) Module #8 (31 to 33) Module #9 (34 to 38) Module #10 (39 to 40)	98.50% (197/200)	0.0150

3	50% (3, 5, 7, 8, and 10)	50% (1, 2, 4, 6, and 9)	4 (60, 37, 220, 169) 5 (84, 106, 155, 187, 182) 5 (33, 222, 144, 23, 123) 5 (199, 85, 38, 78, 103) 5 (63, 143, 89, 191, 93) 5 (122, 189, 135, 95, 181) 5 (91, 194, 227, 119, 130) 3 (188, 124, 238) 5 (44, 105, 217, 102, 199) 5 (114, 129, 24, 140, 208)	Module #1 (1 to 2) Module #2 (3 to 7) Module #3 (8 to 10) Module #4 (11 to 16) Module #5 (17 to 21) Module #6 (22 to 23)Module #7 (24 to 27) Module #8 (28 to 31) Module #9 (32 to 35) Module #10 (36 to 40)	98% (196/200)	0.0200

4	50% (3, 4, 7, 9, and 10)	50% (1, 2, 5, 6 and 8)	5 (52, 173, 68, 176, 133) 5 (143, 202, 54, 67, 55) 5 (82, 142, 191, 47, 183) 5 (205, 115, 95, 143, 218)5 (95, 142, 73, 47, 117) 5 (182, 86, 87, 113, 102) 5 (40, 115, 98, 95, 120) 5 (196, 181, 82, 69, 154) 5 (97, 117, 142, 216, 65) 5 (153, 155, 91, 48, 124)	Module #1 (1 to 3) Module #2 (4 to 6) Module #3 (7 to 9) Module #4 (10 to 13) Module #5 (14 to 15) Module #6 (16 to 22) Module #7 (23 to 27) Module #8 (28 to 31) Module #9 (32 to 35) Module #10 (36 to 40)	99% (198/200)	0.0100

5	50% (2, 3, 5, 8, and 9)	50% (1, 4, 6, 7, and 10)	5 (128, 150, 50, 26, 73) 5 (145, 149, 49, 69, 58) 5 (129, 58, 124, 86, 70) 5 (127, 69, 126, 139, 69) 5 (33, 174, 146, 137, 218) 5 (137, 95, 232, 187, 97) 5 (101, 104, 158, 66, 95) 5 (142, 207, 48, 140, 51) 5 (79, 157, 191, 129, 222) 5 (199, 102, 148, 103, 49)	Module #1 (1 to 2) Module #2 (3 to 4) Module #3 (5 to 13) Module #4 (14 to 18) Module #5 (19 to 20) Module #6 (21 to 25) Module #7 (26 to 30) Module #8 (31 to 33) Module #9 (34 to 35) Module #10 (36 to 40)	98% (196/200)	0.0200

**Table 10:** Comparison of results (test #2, ORL).

Method	Best	Average	Worst
Azami et al. [43]	96.50%	95.91%	95.37%
Ch'Ng et al. [3]	96.5%	94.75%	94%
Sánchez et al. [38]	99%	98.30%	98%
Sánchez et al. [39]	98.43%	97.59%	94.55%
Proposed GWO	99%	98.50%	98%

**Table 11:** The results for iris database.

Trial	Images		Number of hidden layers and number of neurons	Persons per module	Rec. rate	Error
	Training	Testing				
1	79% (1, 2, 3, 5, 6, 8, 10, 11, 12, 13, and 14)	21% (4, 7, and 9)	5 (133, 205, 93, 203, 184) 4 (112, 198, 134, 97) 5 (39, 159, 68, 76, 119) 2 (158, 148) 5 (183, 139, 135, 51, 72) 4 (224, 168, 148, 195) 5 (152, 170, 65, 47, 55) 5 (114, 218, 162, 85, 107) 3 (86, 205, 172)	Module #1 (1 to 15) Module #2 (16 to 22) Module #3 (23 to 34) Module #4 (35 to 45) Module #5 (46 to 47) Module #6 (48 to 49) Module #7 (50 to 64) Module #8 (65 to 74) Module #9 (75 to 77)	99.57% (230/231)	0.0043

2	75% (2, 3, 4, 5, 6, 8, 9, 10, 12, 13, and 14)	25% (1, 7, and 11)	5 (97, 66, 149, 117, 144) 5 (69, 210, 77, 70, 203) 4 (159, 102, 153, 152) 5 (35, 171, 134, 124, 101) 3 (167, 166, 169) 5 (198, 64, 80, 176, 131) 3 (81, 80, 227) 4 (106, 114, 89, 148)	Module #1 (1 to 4) Module #2 (5 to 15) Module #3 (16 to 23) Module #4 (24 to 31) Module #5 (32 to 46) Module #6 (47 to 58) Module #7 (59 to 62) Module #8 (63 to 77)	100% (231/231)	0

6	76% (1, 2, 3, 4, 5, 6, 8, 9, 12, 13 and, 14)	24% (7, 10, and 11)	4 (73, 210, 138, 49) 5 (119, 161, 63, 96, 112) 3 (180, 135, 77) 5 (124, 164, 177, 216, 94) 5 (129, 123, 215, 88, 100) 5 (65, 89, 69, 144, 80) 5 (67, 110, 112, 200, 134) 3 (86, 72, 160)	Module #1 (1 to 3) Module #2 (4 to 13) Module #3 (14 to 30) Module #4 (31 to 40) Module #5 (41 to 51) Module #6 (52 to 60) Module #7 (61 to 65) Module #8 (66 to 77)	99.57% (230/231)	0.0043

7	78% (1, 2, 3, 4, 5, 6, 7, 8, 10, 11, and 13)	22% (9, 12, and 14)	5 (168, 99, 94, 156, 175) 4 (90, 122, 124, 122) 5 (129, 32, 159, 174, 50) 4 (218, 93, 237, 71) 5 (117, 36, 167, 143, 52) 5 (135, 60, 226, 140, 112) 5 (169, 117, 95, 36, 96) 5 (97, 71, 225, 147, 176) 3 (162, 170, 139)	Module #1 (1 to 4) Module #2 (5 to 16) Module #3 (17 to 20) Module #4 (21 to 37) Module #5 (38 to 46) Module #6 (47 to 51) Module #7 (52 to 71) Module #8 (72 to 73) Module #9 (74 to 77)	99.57% (230/231)	0.0043

11	78% (1, 2, 3, 4, 5, 6, 7, 8, 10, 13, and 14)	22% (9, 11, and 12)	5 (86, 162, 217, 168, 168) 4 (167, 189, 62, 193) 5 (115, 53, 154, 105, 79) 3 (62, 89, 134, 87)4 (119, 142, 105, 204) 3 (128, 115, 175, 127)5 (147, 197, 61, 110, 217) 3 (142, 164, 96, 141) 5 (140, 104, 57, 108, 122)	Module #1 (1 to 4) Module #2 (5 to 8) Module #3 (9 to 16) Module #4 (17 to 32) Module #5 (33 to 39) Module #6 (40 to 46) Module #7 (47 to 57) Module #8 (58 to 68) Module #9 (69 to 77)	100% (231/231)	0

**Table 12:** Comparison of results (iris).

Method	Best	Average	Worst
Sánchez and Melin [44]	99.68%	98.68%	97.40%
	0.0032	0.0132	0.0260
Sánchez et al. [37]	99.13%	98.22%	96.59%
	0.0087	0.0178	0.0341
Proposed GWO	100%	99.31%	98.70%
	0	0.0069	0.0130

**Table 13:** Databases setup.

Database	Number of persons	Max. number of images per person		Image size (pixels)
		Training	Testing	
Ear	77	3	3	132 × 91
ORL	40	9	9	92 × 112
FERET	200	6	6	100 × 100
Iris	77	13	13	21 × 21

**Table 14:** The summary of results (proposed method).

Method	Number of images for training	Recognition rate		
		Best	Average	Worst
Proposed method (ear database)	3 (up to 80%)	100%	100%	100%
Proposed method (ear database)	2 (up to 50%)	96.75%	96.15%	95.45%
Proposed method (ORL database)	8 (up to 80%)	100%	100%	100%
Proposed method (ORL database)	5 (up to 50%)	99%	98.50%	98.50%
Proposed method (FERET database)	(up to 80%)	98%	92.63%	88.17%
Proposed method (iris database)	(up to 80%)	100%	99.31%	98.70%

**Table 15:** Table of comparison of optimized results (ear database).

Method	Number of images for training	Recognition rate		
		Best (%)	Average (%)	Worst (%)
Sánchez and Melin [7] (ANN)	3	100%	96.75%	—
Melin et al. [45] (MNN)	3	100%	93.82%	83.11%
Sánchez and Melin [7] (MGNN)	3	100%	99.69%	93.5%
Sánchez et al. [38] (FA)	3	100%	99.89%	98.05%
Proposed method (MGNN)	3	100%	100%	100%
Sánchez and Melin [7] (ANN)	2	96.10%	88.53%	—
Sánchez and Melin [7] (MGNN)	2	98.05%	94.81%	79.65%
Sánchez et al. [38] (FA)	2	97.40%	96.82%	95.45%
Proposed method (MGNN)	2	96.75%	96.15%	95.45%

**Table 16:** Table of cross-validation results (ear database).

Experiment 1	Experiment 2	Experiment 3	Experiment 4	Average
100%	100%	94.81%	93.51%	97.07%

**Table 17:** Table of comparison of optimized results (ORL database).

Method	Images for training	Recognition rate		
		Best (%)	Average (%)	Worst (%)
Mendoza et al. [4] (FIS)	8	97.50%	94.69%	91.50%
Sánchez et al. [38] (FA)	8	100%	100%	100%
Sánchez et al. [39] (MGNNs + complexity)	8	100%	99.27%	98.61%
Proposed method	8	100%	100%	100%
Azami et al. [43] (CGA + PCA)	5	96.5%	95.91%	95.37%
Ch'Ng et al. [3] (PCA + LDA)	5	96.5%	94.75%	94%
Sánchez et al. [38] (FA)	5	99%	98.30%	98%
Sánchez et al. [39] (MGNNs + complexity)	5	98.43%	97.59%	94.55%
Proposed method	5	99%	98.5%	98%

**Table 18:** Table of cross-validation results (ORL database).

Experiment 1	Experiment 2	Experiment 3	Experiment 4	Experiment 5	Average
95.42%	94.58%	96.67%	97.92%	97.92%	96.50%

**Table 19:** Table of comparison of optimized results (FERET database).

Method	Number of persons	Number of images	Recognition rate
Wang et al. [46] (SIFT)	50	7	86%
Proposed method	50	7	98%
Wang et al. [46] (SIFT)	100	7	79.7%
Proposed method	100	7	92.33%
Wang et al. [46] (SIFT)	150	7	79.1%
Proposed method	150	7	92%
Wang et al. [46] (SIFT)	200	7	75.7%
Proposed method	200	7	88.17%

**Table 20:** Table of cross-validation results (FERET database).

Number of persons	Experiment 1	Experiment 2	Experiment 3	Experiment 4	Experiment 5	Average
50	93.33%	95.33%	94.00%	94.67%	94.67%	94.40%
100	83.67%	88.33%	89.00%	91.33%	92.00%	88.87%
150	79.78%	86.44%	87.78%	90.22%	89.33%	86.71%
200	76.17%	83.00%	82.83%	84.50%	85.83%	82.47%

**Table 21:** Table of comparison of optimized results (iris database).

Method	Images for training	Recognition rate		
		Best (%)	Average (%)	Worst (%)
Sánchez and Melin [44] (HGA)	Up to 80%	99.68%	98.68%	97.40%
Sánchez et al. [37] (FA)	Up to 80%	99.13%	98.22%	96.59%
Proposed method	Up to 80%	100%	99.31%	98.70%

**Table 22:** Table of cross-validation results (iris database).

Experiment 1	Experiment 2	Experiment 3	Experiment 4	Experiment 5	Experiment 6	Average
98.27%	99.13%	98.27%	96.97%	97.84%	96.97%	97.91%

**Table 23:** Values of ear database* (test #1).*

Method	*N*	Mean	Standard deviation	Error standard deviation of the mean	Estimated difference	*t*-value	*P* value	Degree of freedom
Sánchez and Melin [7] (MGNN)	30	0.0030	0.0121	0.0022	0.003	1.38	0.1769	29
Proposed method	30	0	0	0				

Sánchez et al. [38] (MGNN)	30	0.00108	0.00421	0.00077	0.001082	1.41	0.169	29
Proposed method	30	0	0	0				

**Table 24:** Values of ORL database* (test #1).*

Method	*N*	Mean	Standard deviation	Error standard deviation of the mean	Estimated difference	*t*-value	*P* value	Degree of freedom
Mendoza et al. [4] (MG + FIS2)	4	94.69	2.58	1.3	−5.31	−4.12	0.026	3
Proposed method	4	100	0	0				

Sánchez et al. [39] (MGNNs + complexity)	5	99.27	0.676	0.30	−0.73	−2.42	0.072	4
Proposed method	5	100	0	0				

**Table 25:** Values of FERET database.

Method	*N*	Mean	Standard deviation	Error standard deviation of the mean	Estimated difference	*t*-value	*P* value	Degree of freedom
Wang et al. [46] (SIFT)	4	80.13	4.29	2.1	−12.50	−4.24	0.00547	6
Proposed method	4	92.63	4.05	2.0				

**Table 26:** Values of iris database.

Method	*N*	Mean	Standard deviation	Error standard deviation of the mean	Estimated difference	*t*-value	*P* value	Degree of freedom
Sánchez and Melin [44]	20	98.68	0.779	0.17	−0.624	−3.18	0.0035	29
Proposed method	20	99.30	0.407	0.091				

Sánchez et al. [37]	20	98.22	0.758	0.17	−1.083	−5.62	1.8623*E* − 06	38
Proposed method	20	99.30	0.407	0.091				

**Table 27:** Values of ear* database (test #2).*

Method	*N*	Mean	Standard deviation	Error standard deviation of the mean	Estimated difference	*t*-value	*P* value	Degrees of freedom
Sánchez and Melin [7] (MGNN)	30	0.0518	0.0345	0.0063	0.01328	2.09	0.045	29
Proposed method	30	0.03853	0.00449	0.00082				

Sánchez et al. [38] (FA)	30	0.03182	0.00462	0.00084	−0.00671	−5.70	4.1926*E* − 07	57
Proposed method	30	0.03853	0.00449	0.00082				

**Table 28:** Values of *ORL database (test #2).*

Method	*N*	Mean	Standard deviation	Error standard deviation of the mean	Estimated difference	*t*-value	*P* value	Degrees of freedom
Azami et al. [43] (CGA + PCA)	5	95.91	0.409	0.18	−2.590	−8.96	1.9091*E* − 05	8
Proposed method	5	98.50	0.500	0.22				

Ch'Ng et al. [3] (PCA + LDA)	4	94.75	1.19	0.60	−3.750	−5.90	0.004	3
Proposed method	5	98.50	0.500	0.22				

Sánchez et al. [38] (FA)	5	98.30	0.447	0.20	−0.20	−0.67	0.523	8
Proposed method	5	98.50	0.500	0.22				

Sánchez et al. [39] (MGNNs + complexity)	5	97.59	1.71	0.76	−0.94	−1.15	0.314	4
Proposed method	5	98.50	0.500	0.22				
